# 3D-Printed Lithium-Ion Battery Electrodes: A Brief Review of Three Key Fabrication Techniques

**DOI:** 10.3390/ma17235904

**Published:** 2024-12-02

**Authors:** Alexander A. Pavlovskii, Konstantin Pushnitsa, Alexandra Kosenko, Pavel Novikov, Anatoliy A. Popovich

**Affiliations:** Institute of Machinery, Materials and Transport, Peter the Great Saint Petersburg Polytechnic University, Politechnicheskaya ul. 29, 195251 Saint Petersburg, Russia

**Keywords:** 3D-printed batteries, lithium-ion batteries, inkjet printing, 3D printing

## Abstract

In recent years, 3D printing has emerged as a promising technology in energy storage, particularly for the fabrication of Li-ion battery electrodes. This innovative manufacturing method offers significant material composition and electrode structure flexibility, enabling more complex and efficient designs. While traditional Li-ion battery fabrication methods are well-established, 3D printing opens up new possibilities for enhancing battery performance by allowing for tailored geometries, efficient material usage, and integrating multifunctional components. This article examines three key 3D printing methods for fabricating Li-ion battery electrodes: (1) material extrusion (ME), which encompasses two subcategories—fused deposition modeling (FDM), also referred to as fused filament fabrication (FFF), and direct ink writing (DIW); (2) material jetting (MJ), including inkjet printing (IJP) and aerosol jet printing (AJP) methods; and (3) vat photopolymerization (VAT-P), which includes the stereolithographic apparatus (SLA) subcategory. These methods have been applied in fabricating substrates, thin-film electrodes, and electrolytes for half-cell and full-cell Li-ion batteries. This discussion focuses on their strengths, limitations, and potential advancements for energy storage applications.

## 1. Introduction

Amidst technological advancements, renewable energy sources such as wind and solar power have emerged as cost-effective alternatives to fossil fuels. However, their natural intermittency requires advanced storage solutions to ensure a consistent energy supply. Rechargeable batteries are currently the most viable option to address this challenge. Significant global efforts have been made to exploit sustainable and renewable resources in recent decades. This has led to ongoing investigations into new energy harvesting and storage devices with advanced electrode materials and architectures [1,2,3,4].

The anticipated growth of the Internet of Things (IoT), wearables, portable microelectronics, medical implants, and smart sensors, combined with increasing demands for the sustainability and security of humanity, has further fueled the development of advanced microscale electrochemical energy storage (EES) devices. These devices are envisioned to provide high stability and performance while remaining lightweight, adaptable, and customizable [5,6,7]. To meet these evolving demands, EES development strategies focus on three key aspects: the adaptable synthesis of active electrode materials, microstructural optimization, and the high resolution of manufactured devices. Conventional fabrication methods for energy storage devices have limitations in controlling electrode geometry, developing solid-state electrolytes (SSEs), and optimizing cell packaging. These issues often lead to subpar ion transport and decreased performance.

Moreover, creating flexible and stretchable batteries requires electrode designs that balance electrochemical performance and mechanical durability during deformation. Although 2D electrode designs improve charge rates by maximizing surface area, they can be at odds with the compactness required for portable devices. In contrast, 3D architectures offer shorter diffusion pathways and improved energy transport, making them a promising alternative.

Among rechargeable batteries, lithium-ion batteries (LIBs) remain the leading EES technology for microelectronics, accounting for about 75% of the global battery market. This dominance is attributed to their efficiency, versatility, rechargeability, and high gravimetric and volumetric energies [8]. LIBs are suitable for various applications, from small consumer electronic devices like laptops and smartphones to large-scale systems such as electric vehicles (EVs) and grid-scale stationary storage [9,10,11]. Nevertheless, LIBs encounter significant challenges, including limited power density for EVs and rigidity that hampers their use in flexible, wearable electronics.

Traditionally, commercial LIBs are designed in fixed shapes such as cylindrical, prismatic, coin, and pouch formats. In these designs, the battery components are usually arranged in rolled or layered planar sheets [12]. However, specialized applications in wearable electronics [13,14], automotive systems, and aerospace devices [15,16] require LIBs that can be tailored into custom geometries. For instance, LIBs integrated into a watchband can power an electronic watch [17], eliminating the need for coin cell installation or replacement. To meet these emerging needs, it is necessary to develop LIBs in flexible, non-standard shapes that incorporate packaging, assembly, and novel manufacturing techniques. To date, the most effective method for creating customizable, freeform LIBs is through additive manufacturing (AM), commonly known as 3D printing [18,19,20,21]. This layer-by-layer, on-demand fabrication approach offers the flexibility needed to design complex 3D LIBs in various shapes.

The typical tape-casting composite positive electrodes for LIBs consist of active materials (60–95 wt. %), conductive additives (3–30 wt. %), and a binder or polymer matrix (2–25 wt. %) [22,23]. For high-performance LIBs, most researchers commonly use a weight ratio of 8:1:1 for these components [24]. The active material stores and releases lithium ions during the charge and discharge cycles. Common active materials include lithium cobalt oxide (LiCoO_2_), lithium iron phosphate (LiFePO_4_), and lithium nickel manganese cobalt oxide (NMC). These materials play a crucial role in the electrochemical process of batteries. Conductive additives such as carbon black (CB), graphite, or carbon nanotubes (CNTs) enhance the electrical conductivity of the electrodes, ensuring efficient electron transport [25]. The binder, such as polyvinylidene fluoride (PVDF) or water-soluble alternatives like sodium salt of carboxymethyl cellulose (CMC) and styrene-butadiene rubber (SBR), provides mechanical stability [26]. It binds the active material and conductive additives together while adhering the composite to the current collector.

Printed electrodes for Li-ion batteries demonstrate significant deviations in electrode composition compared to traditional tape-casting LIB electrodes, mainly due to the need to accommodate the requirements of additive manufacturing processes. As mentioned above, conventional electrodes for LIBs are often optimized with a weight ratio of 8:1:1 for the electrode components in high-power applications. In contrast, 3D-printed electrodes frequently involve higher polymer and binder content, improving printability and mechanical properties but reducing the active material and conductive additive fractions, lowering electrochemical performance. For instance, Maurel and coworkers [27] developed graphite–polylactic acid composite-based filaments for LIB 3D-printing with a composition of 49/5/33/13 wt. % (graphite/carbon black/PLA/PEGDME500). The higher polymer content and use of a plasticizer facilitated extrusion but limited active material loading. Hu and coworkers [28] used thermoplastic polyurethane (TPU) as the polymer matrix in a filament loaded with LFP or LTO active materials, achieving a TPU/active material/conductive additive ratio of 50/44/6 wt. %. The flexibility and durability offered by TPU improved mechanical integrity but reduced the fraction of electrochemically active components. Similarly, Foster and coworkers [29] reported that a PLA/graphene filament with 92/8 wt. % showed poor electrochemical performance due to insufficient conductivity, as graphene was employed as an electroactive material and a conductive additive within a high-polymer content matrix.

Further, an experimental composite photosensitive resin prepared by Martinez and her coworkers [30] incorporated Genesis base resin, LiCoO_2_, and C45 as a conductive additive in a 95:4.7:0.3 wt. % ratio. As far as we know, this work represents the maximum amount of polymer matrix in printed electrodes reported to date. This resin allowed the printing of complex 3D structures via the vat photopolymerization technique, attributed to the high loading of the polymeric matrix, but sacrificed electrochemical performance due to the reduced quantities of active material and conductive additives. A thorough premixing step using a mortar ensured homogeneous dispersion of components, emphasizing the trade-off between achieving structural printability and maintaining electrochemical functionality. These studies underline a common trade-off in printed electrodes: prioritizing printability and structural integrity necessitates higher polymer and binder content, compromising the high active material loading typical of traditional electrodes. While printed electrodes demonstrate promise for innovative designs and flexibility, significant optimization is required to bridge the performance gap compared to conventional counterparts.

The majority of reported 3D-printed Li-ion systems to date consist of half-cell Li-ion batteries utilizing electrodes primarily composed of materials like graphene [29,31,32], graphite [27,33,34,35], Li_4_Ti_5_O_12_ [36], SnO_2_ [37], MnO_2_ [38], and Si [39] as anodes, along with LiFePO_4_ (LFP) [36,40,41] and LiCoO_2_ (LCO) [42,43] as cathodes. Only a few studies have focused on full-cell 3D-printed Li-ion electrodes, mainly combining LFP cathodes with LTO anodes [44,45].

In this review, we explore three key representative 3D printing techniques specifically applied in the fabrication of LIBs: material extrusion (ME), which encompasses two subcategories—fused deposition modeling (FDM), also referred to as fused filament fabrication (FFF), and direct ink writing (DIW); material jetting (MJ), including inkjet printing (IJP) and aerosol jet printing (AJP) methods; and vat photopolymerization (VAT-P), which includes the stereolithography (SLA) technique. These three techniques were selected as the focus of this review because they represent fundamentally distinct and versatile categories of additive manufacturing technologies, each with unique strengths and capabilities that address critical challenges in Li-ion battery design and fabrication. ME is well-suited for bulk deposition and large-scale structural components, MJ offers precision in depositing functional materials without polymer binders, and VAT-P excels in high-resolution patterning for intricate structures. Each of these 3D printing methods has shown significant promise for manufacturing battery electrode materials for LIBs. Although they have unique strengths and limitations, their combined capabilities offer significant potential for advancing high-performance Li-ion battery design [46,47].

In addition to the three main methods discussed in this article—ME, MJ, and VAT-P—other 3D printing techniques are currently being researched for their potential in energy storage applications. These include laminated object manufacturing (LOM) [48,49], binder jetting (BJ) [50], and powder bed fusion (PBF) techniques, the last of which consists of two subcategories: selective laser sintering (SLS) [51] and selective laser melting (SLM) [52,53]. These techniques enable the creation of complex 3D frameworks and hierarchical nanostructures, which improve the electrochemical performance of energy storage devices by maximizing charge carrier mobility and increasing the electroactive surface area of 3D-printed electrode materials. While other techniques also show potential, ME, MJ, and VAT-P are prioritized for their prominence in the literature and their ability to integrate electrochemical functionality with structural complexity.

## 2. Three-Dimensional Printing in Battery Manufacturing: A Step Forward from Traditional Methods

**Figure 1:**
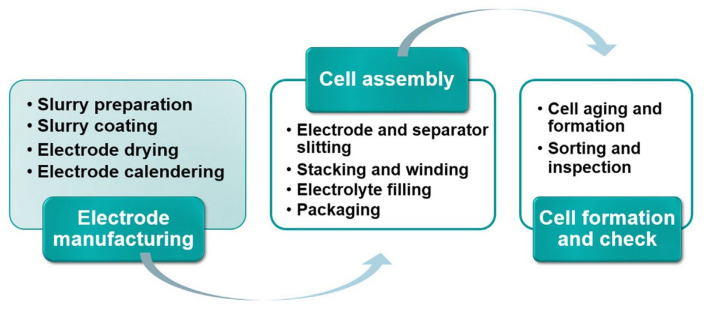
The conventional manufacturing process of commercial lithium-ion batteries. Reprinted from Ref. [54], copyright 2021, with permission from Elsevier.

Manufacturing technologies have become increasingly important with the growing demand for efficient and scalable battery solutions, particularly for LIBs. Traditional methods of LIB fabrication involve a series of complex, time-consuming, and labor-intensive steps, which are illuminated in Figure 1.

These include electrode manufacturing processes such as slurry preparation, slurry coating on metallic foil current collectors, and drying and calendaring of LIB electrodes [55], followed by cell assembly tasks like electrode and separator slitting, stacking and winding, electrolyte filling, and packaging. Finally, cell formation and quality check processes involve cell aging, formation, sorting, and inspection. Each of these steps can introduce variability, impacting the electrochemical performance and consistency of the final product. Additionally, these processes typically require separate equipment, adding to both time and cost.

Traditional LIB production methods generally lack precise control over critical parameters such as areal loading of active materials and microstructure design, which are essential for achieving high performance and efficiency in energy storage. These methods also limit the ability to create complex, customized shapes or structures. For more advanced or structural LIBs, additional high-cost techniques—such as freeze casting or chemical vapor deposition—may be required. However, these approaches are not ideal for large-scale applications due to their high costs, complexity, and material waste.

In contrast, 3D printing offers a more flexible, efficient, and sustainable approach to LIB fabrication [56]. This advancement builds on AM technologies, which construct objects layer by layer directly from Computer-Aided Design (CAD) models, allowing for precise and customizable 3D battery architectures. By enabling the simultaneous preparation of electrodes, electrolytes, current collectors, and even packaging materials within a single setup, 3D printing dramatically simplifies the manufacturing process, reducing time and enhancing consistency [57]. Unlike traditional methods, 3D printing allows for the precise control of microstructures and areal loading, producing high-performance LIBs with customizable shapes and optimized characteristics [58,59,60].

Traditional laminated battery structures often encounter a compromise between energy density and power density. In contrast, 3D battery architectures facilitate electrolyte infiltration among the active components, enhancing the surface-to-volume ratios and reducing the diffusion path for Li-ions. Consequently, 3D cells can deliver both high energy and power densities simultaneously.

Three-dimensional printing technologies offer several advantages over conventional manufacturing methods, making them highly promising for producing structural energy storage devices (ESDs). Firstly, 3D printing enables the simultaneous production of electrodes, electrolytes, current collectors, and packaging materials in one 3D printing machine, simplifying the manufacturing process, improving product consistency, and reducing the time required for ESD preparation [61,62,63,64]. Secondly, 3D printing allows for precise control over the microstructures and shapes of these components through adjustments in printing inks and predesigned programs, enabling the fabrication of structural ESDs with enhanced electrochemical performance [64,65,66,67,68,69]. Additionally, 3D printing provides accurate control over critical factors such as the areal loading of active materials and electrodes’ microstructure, improving electrochemical performance consistency [48,70,71,72,73,74]. Another notable benefit is the efficient use of raw materials, as additive manufacturing (AM) minimizes waste by depositing only the necessary material, addressing inefficiencies inherent to traditional methods.

Moreover, the versatility of 3D printing facilitates the production of LIBs with complex geometries that are otherwise challenging or impossible to achieve with conventional techniques. This capability is advantageous for applications requiring structural or wearable batteries, where integration into unconventional forms and surfaces is essential [75]. By enabling precise control over critical parameters, reducing waste, and allowing for the fabrication of intricate designs, 3D printing technology presents a transformative approach to LIB manufacturing and broadens the possibilities for diverse applications [76]. Given these advantages (Table 1), additive manufacturing holds promise for meeting the evolving demands of the battery industry, facilitating more efficient, sustainable, and innovative LIB production.

## 3. Material Extrusion

Material extrusion (ME) is one of the most accessible and versatile 3D printing techniques. It operates within a three-axis motion stage and utilizes ink or paste ejection through a nozzle to create desired patterns or structures. ME includes two subcategories: fused deposition modeling (FDM) [77], also known as fused filament fabrication (FFF), and direct ink writing (DIW). In Li-ion battery research, ME has been employed to print electrodes by combining polymers with active and conductive materials such as graphite, graphene, carbon black, LTO, and LFP.

### 3.1. Fused Deposition Modeling

Fused deposition modeling involves the deposition of thermoplastic or composite filaments layer-by-layer, making it suitable for creating intricate structures. Unlike inks’ strict rheological property requirements for DIW, FDM imposes fewer material constraints, as it primarily relies on thermoplastic polymers [78]. The working mechanism of FDM shares similarities with DIW, wherein a solid-state filament is extruded through a heated nozzle [79]. During the printing process, the filament is melted, deposited onto a substrate, and solidified upon natural cooling. Despite its widespread use, FDM has found limited application in the 3D printing of EES devices due to plastic filaments’ relatively low electrical conductivity [80]. Currently, there are commercially available graphene PLA filaments which offer superior conductivity and improved mechanical properties. However, the relatively low graphene concentration (8–10 wt. %) within these filaments limits the extent of improvement in their electrical conductivity [29].

Foster and his coworkers [29] were among the first to explore using FDM for printing 3D disc electrode architectures composed of 8% graphene and 92% polylactic acid (PLA). Despite the innovative approach, the low electrical conductivity of PLA limited the specific discharge capacity to only 15.8 mAhg^−1^ at a current density of 40 mAg^−1^ [29]. Their subsequent work focused on enhancing the electrical conductivity by increasing the graphene content within the PLA matrix, which improved specific capacity after chemical pretreatment. The enhanced surface porosity and increased availability of surface graphite led to a significantly improved capacity of 500 mAhg^−1^ at a current density of 40 mAg^−1^ [32].

**Figure 2:**
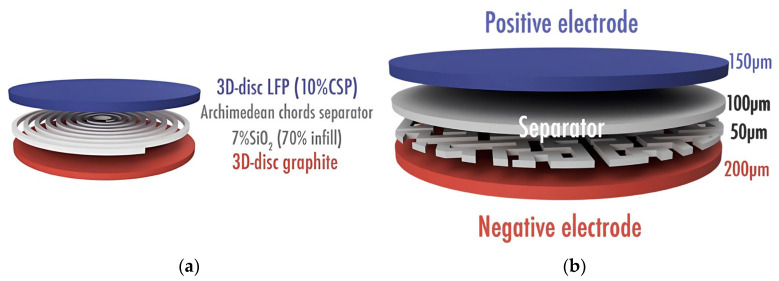
Schematic showing of cells produced through FDM: (**a**) separator layer featuring an Archimedean chord pattern and (**b**) separator layer with a Hilbert curve pattern [35]. Ref. [35] is an open-access article distributed under the terms of the Creative Commons CC BY license, which permits unrestricted use, distribution, and reproduction in any medium provided the original work is properly cited.

In 2019, Maurel and his coworkers [35] utilized FDM to print complete LFP/graphite battery cells with a 3D separator in a single step. They developed 3D-printable PLA/LFP, PLA/SiO_2_, and PLA/graphite filaments with highly loaded active and conductive materials designed to be used as the positive electrode, separator, and negative electrode in an LIB, respectively. These filaments were created to be compatible with FDM 3D printers. Plasticizer PEGDME500 was added to the positive electrode filament to increase the active material (LFP) content as much as possible, enhancing electrochemical performance without compromising the filament’s printability. Films containing Carbon Super P (CSP) showed improved electrical conductivity and specific capacity, as CSP helps connect isolated active material particles within a conductive network, further enhancing performance. The optimized 10%CSP film, which nearly reached its theoretical capacity, was used to produce the corresponding filament for 3D printing. The researchers also tested various battery component designs and found that using Archimedean chords and Hilbert curve patterns as separator layers enhanced electrochemical performance. The enhanced electrochemical performances are attributed to improved soaking of the liquid electrolyte and better impregnation of the electrodes, facilitated by specific separator infill patterns. This adjustment enhances electrolyte distribution, minimizing diffusion limitations and supporting efficient ion transport. Figure 2 shows schematic representations of cells produced by Maurel and coworkers [35].

Study [35] reported the following performance metrics for the printed battery components: 10%CSP 60 μm-thick film of a positive electrode composed of LFP, carbon black, PLA, and a plasticizer in the weight ratio of 49/5/33/13 achieved the best specific capacities of up to 165 mAh/g (C/20) and 162 mAh/g (C/10). However, for the 200 μm-thick 3D-printed positive electrode disc, the capacities dropped to 87 mAh/g (C/20) and 45 mAh/g (C/10) due to the increased thickness (200 µm). The electrodes were tested at current densities of 8.5 mA/g (C/20), 17 mA/g (C/10), 34 mA/g (C/5), and 85 mA/g (C/2). The obtained negative electrode disc fabricated by feeding the filament, consisting of PLA/graphite/carbon black in a weight ratio of 45.9/49.2/4.9, into an FDM 3D printer displayed a reversible capacity of 200 mAh/g at a current density of 18.6 mA/g (C/20) after six charge–discharge cycles. The active material content was 49 wt. % LFP in the printed positive electrode, equivalent to 756 mg/cm^3^, and 49.2 wt. % graphite in the printed negative electrode, equivalent to 773 mg/cm^3^. The study also explored the impact of ceramic additives (SiO_2_) in the separator. Various silica amounts were tested, and it was found that the 7% SiO_2_ sample exhibited the highest ionic conductivity after 10 h of electrolyte soaking, indicating improved performance in terms of ionic transport in the separator.

Similarly, Ragones and his colleagues [36] fabricated an LFP-based cathode using FDM 3D printing with a filament composed of PLA, LFP, and conductive carbon in a weight ratio of 40/50/10. To ensure uniform material dispersion within the filament, the components were pre-mixed using a solvent-based method before being fed into the extruder. However, the resulting cathode’s electrochemical performance remained limited, delivering approximately 60, 50, and 20 mAh/g at current densities of 9, 44, and 88 μA/cm², respectively, which corresponded to less than 50% of the theoretical capacity of LFP. The authors suggested that further filament composition optimization is necessary to realize the full potential of the FDM 3D printing technique. Critical areas for improvement included the electrodes’ composition and morphology, the components’ compatibility and miscibility, and the parameters of the FDM technique. Achieving full utilization of the active material would also require transitioning to a solvent-free process to align with sustainable manufacturing practices.

In 2024, Boudeville and his coworkers published a study [81] demonstrating significant improvements in the electrochemical performance and mechanical properties of FDM-printed LFP electrode discs compared to Maurel’s work [35] based on a single PLA polymer matrix. They proposed the newly developed composite incorporating a co-continuous blend of polar polycaprolactone (PCL) and non-polar polypropylene (PP) polymers, ensuring enhanced mechanical integrity when impregnated with a liquid carbonate electrolyte. SEM and TEM analyses revealed that LFP particles are well-maintained and concentrated in the PP matrix, with carbon fibers forming bridges to create an improved electronic network. At the same time, PCL domains efficiently absorbed and transported the electrolyte. This material distribution, aligned with viscosity-based predictions, highlights the importance of polymer rheology and extrusion parameters. Their base filament (23.7/15.8/55/5.5 wt. % of PP/PCL/LFP/carbon nanofibers) proved too brittle for spooling. To address this disadvantage, 25 wt. % of PP was substituted with a thermoplastic elastomer (ethylene/propylene random copolymer), balancing flexibility, printability, and electrochemical performance. This optimized formulation provided enhanced toughness while maintaining high active material content, demonstrating its potential for scalable 3D-printed electrodes.

Reyes and his coworkers [82] demonstrated the FDM 3D printing of a lithium-ion full cell utilizing lithium titanate (LTO) as the anode material and lithium manganese oxide (LMO) as the cathode material. The process involved formulating composite filaments composed of PLA, LTO, and conductive additives for the anode and PLA, LMO, and conductive additives for the cathode, which were then used as feedstock in an FDM 3D printer. Super P, graphene nanoplatelets, and multi-walled carbon nanotubes (MWCNTs) were conductive additives. The study aimed to determine the optimal component ratios to achieve high electrochemical performance while maintaining the printability of the composite material. A target conductivity of 0.085 mS·cm^−1^ was established, and it was found that up to 30% (*v*/*v*) of solids could be successfully integrated into the PLA matrix without hindering its processability. The results indicated that a conductive material to the active material ratio of 80:20 maximized charge storage capacity. The most effective configuration included lithium titanate and graphene nanoplatelets at the anode and lithium manganese oxide paired with MWCNTs at the cathode, demonstrating the potential of this composite approach to enhance battery performance.

He and his collaborators [33] developed a high-loading 3D Gt@GS (Gt@Gt/SiO) electrode using a modified 3D printing method. This unique structure effectively mitigated volume expansion in all directions and provided a three-dimensional transport network, enhancing ion and electron mobility within the thick electrodes. Consequently, the resulting three-dimensional Gt@GS electrode demonstrated excellent performance as a freestanding material, delivering a high capacity of 3.52 mAh/cm^2^ after 120 charge–discharge cycles at a current density of 3.6 mA/cm^2^, along with perfect cycling stability.

Overall, FDM represents a widely adopted 3D-printing technique for fabricating intricate three-dimensional structures via a layer-by-layer approach, achieved by extruding the targeted active material through a nozzle. In this process, molten metal or plastic filaments are dispensed through a nozzle, which operates across three axes under the guidance of computer-controlled systems [83]. Post-processing steps, including surface treatment methods such as solidification and surface hardening, are employed to achieve the intended structural patterns upon deposition onto the substrate. The solidification phase is governed by fundamental mechanisms like crystallization and chain entanglement [58,65]. Despite its numerous advantages, including low cost, affordability, high resolution, and rapid production speeds, the requirement for surface treatment following material deposition remains a significant limitation to its broader application potential [84].

### 3.2. Direct Ink Writing

Direct ink writing (DIW) is an additive manufacturing technique that enables the direct extrusion of slurry-based inks to produce 3D-printed batteries, a process sometimes referred to as robocasting. This technology, developed in 1996 for creating complex structures of ceramic green bodies, has recently been applied to print various battery components, including negative electrodes, gel electrolytes, positive electrodes, and packaging. The popularity of DIW for electrode fabrication lies in its cost-effectiveness, ease of operation, and material flexibility [85].

The printing process involves preparing viscoelastic inks based on gels that exhibit shear-thinning properties. The design of the printable ink is a critical factor in the DIW technique. Additionally, factors such as ink rheology, printing speed, and resolution significantly influence the outcome of the printing process, allowing resolutions of up to 1 μm to be achieved through parameter optimization. However, a significant challenge that needs addressing is the mechanical stability of the printed films, necessitating substantial advancements in the DIW method [86].

In DIW, inks infused with active materials are deposited directly, driven by extremely high air pressure. Due to the shear-thinning properties of these highly viscous inks, the materials are extruded into semi-solidified and self-supporting filaments as they are formed. This characteristic ensures stability and control over the filament shape during deposition, which is essential for building precise and durable structures layer by layer.

A key advantage of additive manufacturing processes is their ability to print electrodes in virtually any geometry. For instance, both Lacey [87] and Wang [59] have successfully demonstrated the 3D printing of electrodes in mesh and lattice structures, which introduce beneficial macroporosity, improving lithium-ion transport even under high charging and discharging rates. The flexibility in geometric design also makes it possible to create electrodes with high aspect ratios and increased areal capacity—features that are typically challenging to achieve with traditional slurry-casting techniques.

Sun and coworkers [44] pioneered printing high-aspect-ratio, multilayered, interdigitated electrodes for micro lithium-ion batteries (micro-LIBs), achieving high energy and power densities. However, despite these significant advantages, extrusion printing has its challenges. Specifically, ensuring that the inks maintain the necessary composition and rheological properties, as these factors directly impact clogging prevention, adhesion to the substrate, and structural integrity throughout the printing process [88,89].

**Figure 3:**
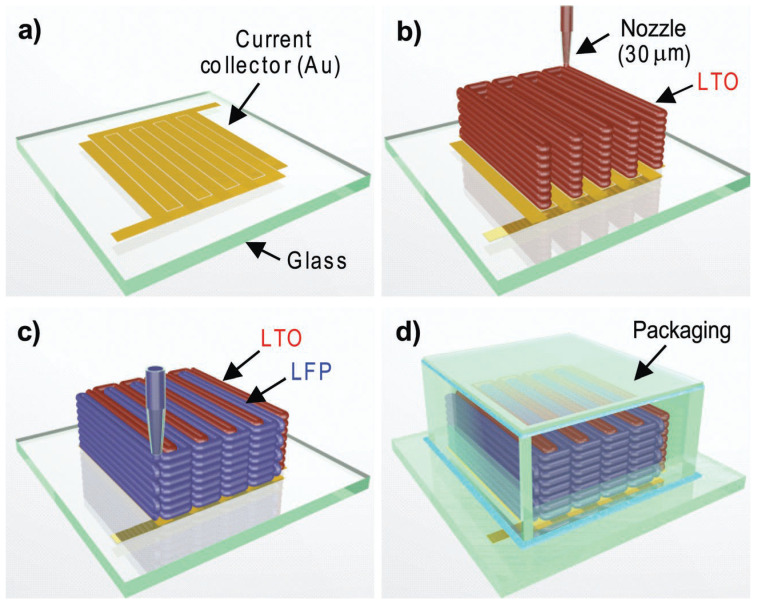
Schematic showing the 3D interdigitated microbattery architecture (3D-IMA) assembled on (**a**) a gold current collector through the sequential printing of (**b**) LTO and (**c**) LFP inks using 30 μm cylindrical nozzles, followed by a sintering process and (**d**) final packaging. Reprinted from Ref. [44], copyright 2013, with permission from WILEY-VCH Verlag GmbH & Co. KGaA, Weinheim, Germany.

Sun and coworkers [44] also reported the fabrication of 3D-printed microbatteries (Figure 3) with interdigitated arrays of LTO anodes and LFP cathodes. These microbatteries achieved an impressive areal energy density of 9.7 J/cm^2^ at a power density of 2.7 mW/cm^2^, showcasing outstanding performance due to the high aspect ratios of the electrodes combined with a compact area footprint.

In 2016, Kohlmeyer and his coworkers [90] developed a positive electrode ink composed of PVDF, LFP, and carbon nanofibers (CNFs) in a 20/40/40 weight ratio using N-methyl-2-pyrrolidone (NMP) as a solvent. The electrochemical performance, tested with a commercial electrolyte containing 1M LiPF_6_ in a 1:1 (vol.) ethylene carbonate/diethyl carbonate (EC/DEC) mixture, showed good functionality for the printed electrodes, delivering 156 mAh/g at a current rate of C/5 and 106 mAh/g at 5C. Four years later, Bao and colleagues [91] employed the same AM technique to develop a flexible battery. Their ink was composed of a PVDF polymer and LFP active materials. However, there were some key differences: they replaced CNFs with multiwalled carbon nanotubes (MWCNTs), a more conductive carbon. They adjusted the ratio to have a higher content of PVDF and a lower content of carbon additive (PVDF/LFP/MWCNTs in a 50/33/17 wt. % ratio). The electrochemical performance resulted in a similar discharge capacity of 154.5 mAh/g at a slightly higher C-rate of C/3 using a similar electrolyte.

DIW has proven helpful in fabricating electrodes for microenergy storage systems. For example, Izumi and his coworkers [92] utilized the DIW technique to print a cathode electrode made from a mixture of lithium titanium oxide as the active material and polyvinylidene difluoride as a binder in NMP solvent, which was then used in lithium-ion energy storage applications. They reported that the cell featuring a 3D patterned electrode demonstrated significantly improved charge and discharge capacities compared to a conventional cell with a flat LTO electrode, particularly at high rates. Furthermore, it was confirmed that the cycle performance of the cell with the 3D patterned electrode was equal to or exceeded that of the traditional flat electrode.

## 4. Material Jetting

### 4.1. Inkjet Printing

Inkjet printing (IJP) is a technique within the material jetting (MJ) category that enables the precise deposition of individual micro-droplets of ink on specific substrate areas to build detailed structures [93]. Initially predominant in graphic arts [94], IJP has expanded into producing printed electronics, including battery electrodes, light-emitting devices, supercapacitors, sensors, thin-film transistors, detectors, and solar cells [38,95,96,97,98]. Over the last decade, IJP’s efficiency and adaptability have brought it to the forefront of industrial applications [99].

A primary advantage of IJP lies in its digital, contact-free deposition approach, which allows nearly limitless design possibilities and quick transitions between configurations without extensive setup. This flexibility supports multi-material and multilayer designs, making it highly suitable for complex applications [100]. Precise control over ink volume minimizes material waste and helps ensure resource efficiency, while the formation of fine droplets results in high-resolution patterns [101,102]. Additionally, its noncontact deposition technique makes IJP compatible with diverse substrates, regardless of their surface properties.

Specifically in energy storage, IJP has shown potential in fabricating high-performance Li-ion battery electrodes. Operating as a binder-free process, IJP directly deposits active materials and conductive additives, enhancing the device’s electrical performance. Despite its advantages, IJP does face some limitations. The limitations of inkjet printing (IJP) arise from the need for parameter optimization, specific design requirements, and challenges with droplet formation. Common issues associated with the IJP technique include nozzle clogging, substrate wetting properties, difficulties achieving film homogeneity, and uniform droplet size [93,103].

Early pioneering work applying IJP to LIBs was carried out by the Shanghai Key Laboratory of Molecular Catalysis and Innovative Materials, with publications in 2006 [37], 2008 [43], and 2009 [96]. These initial studies detailed the formulation of SnO_2_, LTO, and LCO inks, recommending methods like ball milling and ultrasonic baths to ensure a uniform ink consistency. The studies successfully utilized a Canon BC-03 cartridge (Canon Inc., Tokyo, Japan) for the printing process; however, they did not disclose critical ink properties such as viscosity, surface tension, or stability, which are essential for consistent printing performance.

In 2015, Gu and his coworkers [40] utilized the IJP technique to produce LFP cathode thin films, preparing the ink by bath sonication and using centrifugation to remove larger particles before printing. The ink’s viscosity was set at 13 mPa·s to match the optimal requirements for the Dimatix-2800 printer (Fujifilm Dimatix, Inc., Santa Clara, CA, USA). When comparing electrochemical performance, it was observed that the obtained inkjet-printed cathodes had lower capacities than those produced using tape casting. However, this was likely due to differences in ink formulation versus the paste used in doctor blading, suggesting that the direct comparison may need to be more objective [40].

The same year, Dellanoy and his coworkers explored IJP for LFP cathode [41] and silica-based ionogel electrolyte fabrication [45]. Using a specialized piezoelectric inkjet printer, the authors adhered to specific ink parameters: a 10–12 mPa·s viscosity range, a 28–33 mN/m surface tension, and particle sizes below 200 nm. They tested three formulations with different additives—poly-acrylic-co-maleic acid (PAMA), carboxymethyl cellulose (CMC), and a combination of CMC and Triton X-100—prepared through magnetic stirring and ball milling. Rheological measurements confirmed viscosity and stability at rest (storage and shear moduli). Printability tests indicated that inks containing CMC were unsuitable for ejection, highlighting the critical role of additive selection in achieving optimal printability. The ink containing PAMA demonstrated good printability, likely due to its relatively low viscosity under high shear rates and strong deposition stability. However, this observation is preliminary, and further investigation is needed to elucidate the relationship between ink printability and rheological properties fully.

Additionally, the lower molecular mass of PAMA compared to CMC may significantly enhance the ink’s processability. Electrochemical tests on the inkjet-printed electrode revealed outstanding cyclability and high-rate charge/discharge capabilities. The Dimatix DMP-2800 inkjet printer (Fujifilm Dimatix, Inc., Santa Clara, CA, USA) was employed to fabricate the electrolyte. Although the ionogel’s viscosity was characterized, other critical ink properties were not detailed. The inkjet-printed electrolyte, tested within a complete lithium-ion cell, demonstrated performance levels comparable to those achieved through more costly physical vapor deposition (PVD) techniques, showcasing IJP as a cost-effective alternative [41,45].

**Figure 4:**
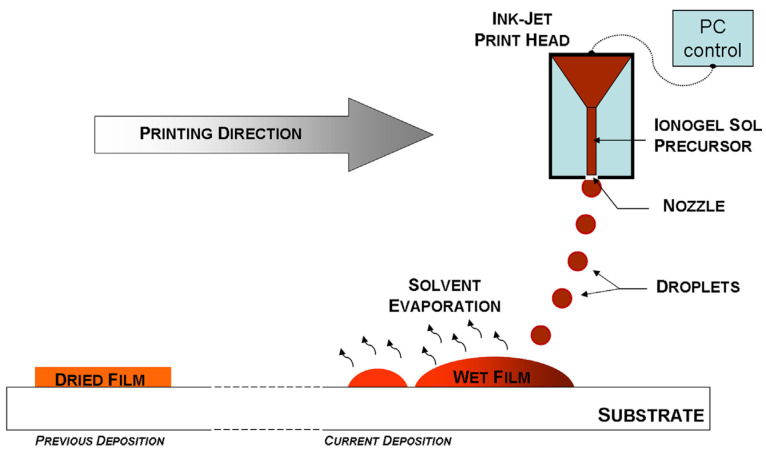
Schematic showing ionogel inkjet printing process. Reprinted from Ref. [45], copyright 2015, with permission from Elsevier.

Overall, Delannoy and his coworkers demonstrated using IJP (Figure 4) to print silica-based ionogels onto iron phosphate and titanate electrodes. Their porous composite electrodes, combined with a 3D-printed ionogel electrolyte, resulted in a full Li-ion cell with an aerial capacity of 300 mAh/cm^2^ for up to 100 cycles [45]. These results highlight the feasibility of using MJ to fabricate efficient, safe, and cost-effective Li-ion batteries with excellent ionic conductivity.

Lawes employed an HP Deskjet 2540 inkjet printer (Hewlett-Packard, Mississauga, ON, Canada) to deposit TiO_2_ [39] and Si [104] anodes, preparing both inks by sonication and adjusting the viscosity to the manufacturer-recommended value of 10 mPa·s. In Lawes’ studies, three binder types were evaluated for the TiO_2_ electrode: polyvinylidene fluoride (PVDF), polyvinylpyrrolidone (PVP), and poly(3,4-ethylenedioxythiophene) polystyrene sulfonate (PEDOT:PSS). Only PEDOT:PSS met the printability requirements, as PVDF and PVP formulations led to agglomeration and nozzle clogging. For the Si anode, PEDOT:PSS, PVP, carboxymethyl cellulose (CMC), and Na-alginate were tested as binders. The findings demonstrated that each ink had good dispersion and printability. Anodes formulated with PEDOT:PSS achieved the highest stability, attributed to its electrical conductivity and ability to accommodate reversible deformation during electrode cycling [104].

Kushwaha’s research group [105] demonstrated the feasibility of using inkjet printing to create environmentally friendly graphene film for as a high-performance anode for LIBs. The study highlighted that ink stability and viscosity are essential for successful printability; however, the article did not comprehensively analyze rheological properties.

Using inkjet printing, Maximov and his coworkers [106] developed an NMC cathode for LI-ion microbatteries. The ink was dispersed using an ultrasonic bath, with larger agglomerates removed by centrifugation. N-methyl-2-pyrrolidone (NMP), commonly used in cathode fabrication, was selected as the solvent. Rheological properties, including viscosity and surface tension, were optimized to meet printer requirements (8–10 mPa·s and 28–32 mN/m, respectively). They investigated the effects of additives such as ethylene glycol, diethylene glycol, and propylene glycol on the slurry’s characteristics, confirming stability through ζ-potential measurements. Although the authors identified optimal conditions for printing, they did not provide results regarding the performance or characteristics of the inkjet-printed layers [106].

**Figure 5:**
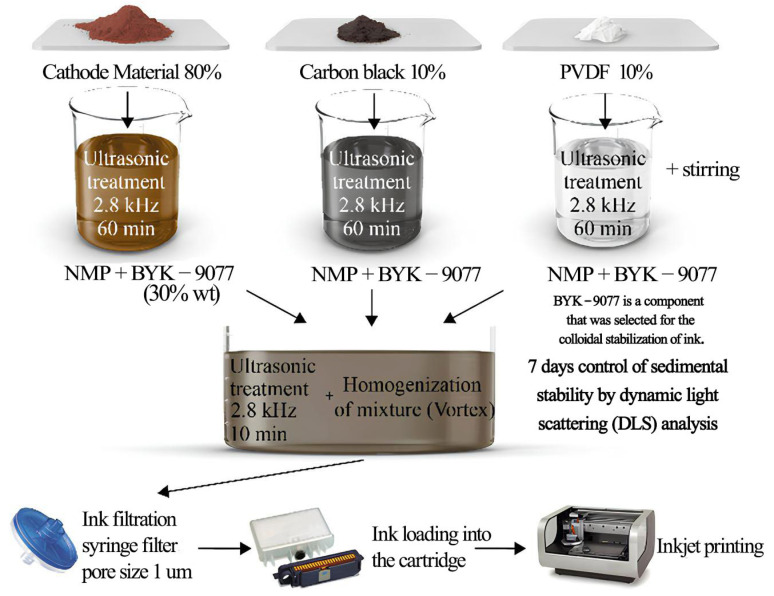
Technological flow chart of ink preparation used by Kolchanov and his coworkers. Reprinted from Ref. [103], copyright 2013, with permission from WILEY−VCH Verlag GmbH & Co. KGaA, Weinheim.

In another work, Kolchanov and his coworkers [103] developed NMC-based cathode inks, carefully optimizing fundamental rheological properties—viscosity, surface tension, and contact angle—to suit inkjet printing requirements. They adjusted the dispersing agent concentration and assessed the sedimentation stability via centrifugation (Figure 5). Printability tests confirmed the generation of a stable droplet, enabling the fabrication of a cathode thin film. Electrochemical performance testing of the inkjet-printed cathode showed values comparable to those of electrodes made using the traditional tape-casting method.

Viviani’s research group [107] studied the impact of carbon additives on the electrochemical performance of inkjet-printed LTO anodes, which they fabricated using a thermal inkjet printer. The rheological measurements were conducted to ensure that the slurries met the printing requirements. Notably, electrodes utilizing carbon nanotubes (CNTs) as conductive agents exhibited the highest specific capacity among the printed films, indicating that CNTs can significantly enhance electrochemical performance and are well-suited for the IJP method.

Wang and his coworkers [108] utilized inkjet printing to create a V_2_O_5_/MXene heterostructure cathode for LIBs. While the inks were found suitable for printing, the study did not provide specific details about their properties. Nonetheless, the printed cathode layers exhibited exceptional electrochemical performance, underscoring the potential of two-dimensional heterostructures in enhancing high-performance battery applications.

In 2016, Fu and his coworkers [109] utilized a polymer composite ink composed of poly(vinylidene fluoride)-co-hexafluoropropylene (PVDF-co-HFP) and Al_2_O_3_ nanoparticles to create a solid-state composite separator and gel electrolyte. By printing this material between GO-based LFP cathodes and LTO anodes arranged in an interdigitated pattern, they achieved a full cell capacity of approximately 100 mAh/g after ten cycles at a current density of 50 mA/g, with the coulombic efficiency reaching nearly 100% after the second cycle [109]. This approach further demonstrated the ability of MJ to produce competitive 3D-printed full-cell Li-ion batteries.

One of the key obstacles in advancing Li-ion batteries designed to function at high voltages (beyond 4.2 V vs. Li/Li^+^) is the corrosion of the cathode current collector. This issue contributes to electrochemical instability and a rise in impedance over time. To tackle this problem, researchers have developed a graphene-based ink using solvent exfoliation, which serves as a protective layer when applied to the current collector. For instance, Kushwaha and his coworkers [110] deposited a thin graphene layer (~268 nm thick) onto aluminum current collectors (AlCCs) through inkjet printing, followed by annealing in an argon atmosphere at 350 °C to enhance the film’s electrical conductivity. The authors precisely controlled the thickness and mass loading of the graphene layer by adjusting the number of printing cycles. When these graphene-coated AlCCs (Gr-AlCCs) were employed for galvanostatic cycling in Li “half” cells with NMC-based cathodes, they demonstrated remarkable cyclic stability. At an upper cut-off voltage of 4.5 V, the Gr-AlCCs retained approximately 90% of their capacity after 100 charge–discharge cycles at a current rate of C/5. In comparison, uncoated AlCCs retained only about 68% capacity under the same conditions.

Although IJP has shown potential for developing 3D structures [111,112] and thin films [113] for LIBs, research in this area remains limited. Table 2 summarizes the studies conducted, focusing on the most relevant details.

While rheological properties are essential for determining ink printability, previous studies have provided only basic measurements or omitted fluid characteristics. Given the complexity of inks used in LIB fabrication, where flow behavior directly affects processability, a deeper exploration of the relationship between rheology and printability is warranted.

### 4.2. Aerosol Jet Printing

**Figure 6:**
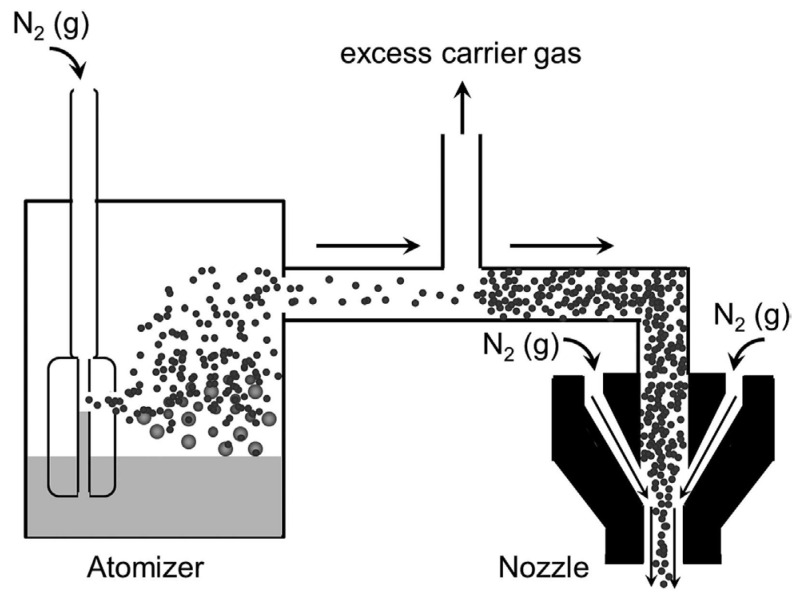
Schematic representation of the aerosol jet deposition process. The process begins within the atomizer, where a carrier gas (illustrated here as N_2_(g)) flows at high velocity across a nozzle submerged in the ink reservoir. The rapid movement of the carrier gas generates a localized region of low pressure, facilitating the formation of aerosolized droplets. Within this system, smaller droplets become entrained in the carrier gas stream, while more significant, heavier droplets are recycled back into the ink reservoir. As the entrained aerosol advances toward the nozzle, the stream undergoes a refinement stage, during which excess carrier gas is removed, resulting in a higher concentration of aerosolized droplets. Upon reaching the nozzle, a secondary sheath gas (also depicted as N_2_(g)) is introduced. This sheath gas plays a critical role in focusing the aerosol stream, ensuring precision and uniformity during deposition. Reprinted from Ref. [2], copyright 2017, with permission from John Wiley and Sons.

Aerosol printing can handle various materials, including polymers, metallic conductors, semiconductors, carbon-based nanomaterials, and energy materials [2]. These materials are dispersed in inks with viscosities from 1 to 1000 cP. In this process, which is illuminated in Figure 6, moderately pressurized air converts the active materials into a fine aerosol mist, enabling highly precise material deposition with feature sizes as small as 10 μm [118].

One distinct advantage of aerosol printing, especially for freeform fabrication, is its ability to deposit on nonplanar substrates with complex surface profiles, such as trenches and wavy structures, with features ranging from tens of microns to millimeters. This flexibility is made possible by the adjustable working distance of 1–5 mm between the deposition nozzle and the substrate [119].

Deiner and coworkers fabricated a 170 μm thick porous cathode using aerosol printing [15]. In work [120], aerosol printing of LFP cathodes and LTO anodes for LIBs was reported. Electrodes with an arbitrary geometry, adjustable thickness, and compatibility with nonplanar substrates were successfully fabricated. The highest achieved areal capacity was approximately 7.1 mAhcm^−2^, at least double that of traditional electrodes. Furthermore, 3D enclosures were created for electrode packaging using fused deposition modeling with polyvinylidene fluoride. The printed electrodes housed in these 3D enclosures retained 78.4% of their capacity after 30 cycles. Customizable LIBs can be developed directly on target objects using these two additive manufacturing processes. For example, a nonplanar LIB was demonstrated conforming to the edge of a block with a specific capacity of 135 mAhg^−1^.

Although aerosol printing provides significant flexibility for electrode design, allowing for customizable geometry, adjustable thickness, and compatibility with non-planar surfaces, these features still need to be fully explored in practical electrode applications.

Summarizing Section 4, it is evident that inkjet and aerosol jet printing have made notable strides in the fabrication of electrochemical devices over the past decade. These techniques have enabled the printing of electrode and electrolyte layers for batteries, supercapacitors, and fuel cells with micro- to mesoscale precision in all dimensions. Printed layers can be functionally graded and deposited onto diverse substrates, offering performance benefits such as thin films, precise electrode patterning, and tailored functionality. Despite these advancements, further research and development are required to fully realize their capabilities for scalable and cost-effective manufacturing of high-performance devices. Addressing challenges such as ink formulation, film deposition, and property optimization across scales will be critical to achieving functional integration and advancing electrochemical device technology.

## 5. Vat Photopolymerization

Vat photopolymerization (VAT-P) is a 3D printing method that uses light to cure photosensitive polymers layer by layer. Although VAT-P has been less explored in the context of Li-ion battery electrode fabrication compared to ME and MJ, it shows promise due to its high resolution, ranging from 100 µm down to 100 nm [121,122], and ability to create exact and complex structures [123]. Despite this capability, it has often been overlooked for electrode fabrication.

Stereolithography (SLA), a subcategory of VAT-P, remains one of the most widely utilized techniques in additive manufacturing due to its precision and versatility. This method employs a photopolymerization reaction triggered by a UV laser to polymerize photocurable resins and form solid polymeric layers. The process involves a flexible resin platform that supports the formation of each layer, which is then repositioned to enable the sequential deposition of subsequent layers. The specific movement of the platform depends on the type of SLA employed: in top-down SLA, the platform is lowered for each consecutive layer, while in bottom-up SLA, it is raised. After repositioning, a blade spreads additional photocurable resin over the preceding layer, preparing it for the subsequent polymerization step. Thanks to its capability to produce highly intricate and complex structures, SLA holds significant potential for applications such as the 3D printing of EES devices [64,70].

Cohen’s research group [67] was among the first to apply VAT-P to print Li-ion battery electrodes. They designed a 3D-printed perforated polymer substrate using SLA technology and fabricated a three-layer structure consisting of an LFP cathode, a LiAlO_2_-PEO membrane, and an LTO anode. This novel approach eliminated the need for metallic current collectors, resulting in a high areal capacity of 400–500 µAh/cm^2^ when cycled between 0.1 and 10 C. While this quasi-solid 3D microbattery exhibited significant capacity decay over time, its areal energy density was three times higher than that of commercial planar thin-film batteries. The authors suggested that further optimization of the printing process and the incorporation of mechanically robust, mass-loaded composites could allow 3D-printed full microbatteries to outperform state-of-the-art thin-film technologies [67].

**Figure 7:**
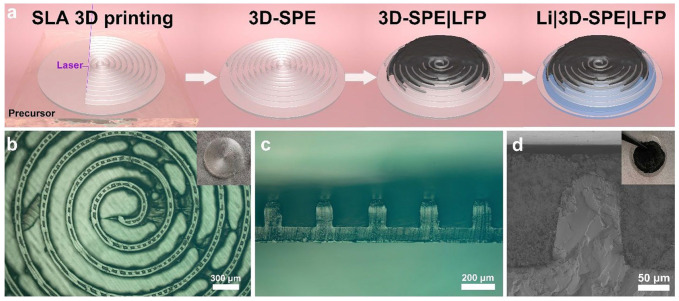
The 3D solid polymer electrolyte created via SLA: (**a**) schematic overview of the cell fabrication process, (**b**) top-view image of the obtained 3D-SPE, (**c**) cross-sectional image highlighting the internal structure, and (**d**) detailed view of the interface between the printed 3D electrolyte and the cathode. Reprinted with permission from Ref. [124], copyright 2020, American Chemical Society.

He and his coworkers [124] made a three-dimensional solid polymer electrolyte (3D-SPE) using stereolithography with poly(ethylene oxide) (PEO) as the electrolyte material. After printing, a cathode slurry containing LFP as the active material was applied to the 3D-SPE via tape-casting, while lithium foil was used as the anode. Figure 7 illuminates the cell preparation process and images of the printed electrolyte.

For comparison, He and his coworkers [124] also fabricated cells with non-structured electrolytes. The 3D architecture enabled higher active material mass loading, leading to a greater specific capacity than the layered structure. Additionally, it achieved improved capacity retention. The authors attributed this enhanced performance to a shortened Li-ion transport pathway between the electrolyte and electrode and better interfacial adhesion during cycling.

Similarly, Sabato and his colleagues [125] successfully developed complex-shaped Li_1.5_Al_0.5_Ge_1.5_P_3_O_12_ (LAGP) full-ceramic electrolytes from glass feedstock using SLA. The printed electrolytes exhibited an ionic conductivity that closely matched that of LAGP fabricated through conventional methods (σ = 6.42 × 10^−5^ S/cm^2^). Moreover, symmetric cells assembled with lithium metal anodes demonstrated stable cycling performance over 250 h, confirming the stability of the designed cell.

Chen and his coworkers [64] employed SLA to fabricate an innovative poly(ethylene glycol) (PEG)-based gel polymer electrolyte containing LiClO_4_ salt. The Li-ion cell was created using this 3D-printed solid electrolyte paired with lithium titanate oxide and lithium iron phosphate as negative and positive electrodes. The study demonstrated that incorporating solvated lithium salt into the polymer matrix enhances lithium-ion transport, particularly within the amorphous regions of the polymer. This incorporation promotes better mobility of the polymer chains and increases ionic conductivity. The electrolyte achieved a high ionic conductivity of 4.8 × 10^−3^ S/cm and a discharge capacity of 1.4 μAh/cm^2^ over two charge–discharge cycles at a current of 5 µA, indicating a positive interaction between the active material and the gel polymer electrolyte. However, when cycling at a higher current (C/5), a gradual decline in capacity was observed, leading to failure by the 10th cycle, likely due to lithium dendrite growth that resulted in short circuits. To address these limitations, the study suggested potential optimizations, including incorporating SiO_2_ into the electrolyte to mitigate dendrite formation and utilizing PEG-based materials in the separator to enhance mechanical stability.

In 2024, Martinez and her coworkers [30] reported a composite UV-sensitive resin developed as a feedstock for a VAT-P system, marking the first successful preparation and additive manufacturing of a resin specifically designed to print a cathode electrode for a conventional LIB. The resin was infused with LiCoO_2_ as the electrochemically active cathode material, complemented by carbon-based additives to enhance conductivity. Challenges arose from the composite’s opacity and high viscosity, which caused light refraction during selective UV curing. After printing, the components underwent thermal post-processing to balance electrochemical performance and mechanical strength. Both sintered and green state 3D-printed cathodes were assembled into half-cell lithium-ion batteries with lithium metal as the reference and counter electrode. These batteries exhibited promising electrochemical cycling results, approaching the performance of commercial LiCoO_2_ cathodes while demonstrating the potential benefits of additive manufacturing, including high-surface-area configurations for improved power performance and shape adaptability.

Overall, SLA offers the unique advantage of enabling truly arbitrary designs compared to ME. Unlike extrusion-based 3D-printing methods, SLA is not constrained by toolpaths or serial extrusion, allowing for fabricating intricate 3D geometries with hollow or carved features on a macroscale [126]. Notably, a graphene-based photocurable resin has been recently synthesized, enabling the printing of high-surface-area macroscale 3D architectures [126,127]. These graphene structures, characterized by their complex designs, exhibit enhanced mechanical properties and electrical conductivity, highlighting their potential for advancing EES device applications.

However, the SLA process has several challenges related to material selection and laser characteristics. These include:

- The lower viscosity of the material, which can affect the stability and integrity of printed layers;
- The light sensitivity of the material, which may pose difficulties during the curing or solidification step when a laser is used;
- The slower scan rate of the laser, which can reduce the speed of the printing process and increase fabrication time;
- The laser beam’s size, which influences the resolution of the printed patterns, thereby affecting the overall quality of the 3D-printed Li-ion batteries.

Together, these issues limit the application of stereolithography in the production of 3D-printed Li-ion batteries, impacting critical factors such as fabrication time, resolution, and material compatibility [128,129,130].

## 6. Performance Comparison, Advantages, and Limitations of the Three Key 3D Printing Techniques

When comparing ME, MJ, and VAT-P technologies, each presents unique advantages and challenges (Table 3). ME is well-suited for printing entire battery assemblies, including current collectors, electrodes, and casings, in a single or multi-step process. However, using plastic polymers like PLA and ABS in ME can limit ionic and electronic conductivity, necessitating the incorporation of conductive additives like graphene or graphite. This need often leads to challenges in optimizing the balance between mechanical strength and electrochemical performance.

Conversely, MJ offers a more direct approach to printing functional active materials without relying on polymer binders, leading to better half-cell and full-cell performance. For instance, MJ-printed LFP cathodes have shown specific capacities as high as 151 mAhg^−1^ at a current density of 15 mAg^−1^ [40], and even carbon-coated LFP electrodes demonstrated a stable capacity of 80 mAhg^−1^ at a rate of 9C (1530 mAg^−1^) over 100 charge–discharge cycles [41]. However, MJ is less versatile when printing complex multi-material objects, as the layer-by-layer deposition process is primarily limited to simple geometries.

While still in its early stages, VAT-P holds significant potential for creating high-resolution, intricately designed battery components. The ability to print fully 3D microbattery structures without metal current collectors represents a breakthrough in battery design. However, challenges remain in improving the mechanical and electrochemical stability of the printed components.

As mentioned above, VAT-P uses a layer-by-layer curing process to solidify a liquid UV–photosensitive resin made from a mixture of polymers and photoinitiators. To create electrochemically active structures, solid or soluble battery materials must also be incorporated [131,132]. However, solid particles, particularly nanoscale ones, can scatter or hinder the absorption of UV light, complicating the curing process. Consequently, exposure time, temperature, layer thickness, and UV light brightness must be carefully optimized to balance printability with electrochemical functionality.

## 7. Conclusions and Outlooks

Overall, 3D printing is poised to revolutionize the fabrication of Li-ion battery electrodes by enabling greater flexibility in design and material selection. Each printing technology—ME, MJ, and VAT-P—offers unique benefits that can be leveraged depending on the desired application. However, challenges related to material conductivity, stability with organic electrolytes, and integrating complex electrode architectures remain. Future advancements in 3D printing for battery applications will likely focus on optimizing material compositions, improving the rheology of deposition, and addressing long-term stability issues.

While each of the abovementioned 3D printing techniques presents unique strengths, the integration of hybrid methods could address limitations in single-method approaches and unlock new possibilities in battery design. From our point of view, hybrid 3D printing methods hold immense potential for revolutionizing battery fabrication by combining the unique advantages of three key 3D printing techniques into a unified process. These approaches enable the integration of diverse materials and structures, allowing for the simultaneous optimization of resolution, material compatibility, and scalability. For instance, hybrid processes can combine material extrusion for bulk electrode deposition, VAT photopolymerization for high-resolution electrolyte layers, and material jetting for intricate current collector patterns. This integration can lead to enhanced device performance by enabling precise control over material placement, microstructure, and interfacial properties.

Moreover, hybrid techniques can address challenges associated with single-method AM approaches, such as limited material versatility or constraints in feature size. By leveraging the complementary strengths of multiple AM processes, it becomes feasible to fabricate complex, multifunctional battery architectures tailored to specific applications, such as high energy density or rapid charge–discharge cycles. Additionally, hybrid systems may allow for in-line post-processing steps, such as curing or sintering, further streamlining the manufacturing workflow.

The adoption of hybrid 3D printing for energy storage devices could pave the way for breakthroughs in both performance and design flexibility, facilitating the creation of next-generation batteries that are lighter, more efficient, and adaptable to various form factors. This approach represents a promising research direction that could significantly advance the field of battery manufacturing.

## Figures and Tables

**Table 1:** Comparing 3D printing and conventional battery fabrication techniques.

Technique	Characteristics in Battery and Component Fabrication
Traditional Methods	Complex, multi-step process Time-consuming and labor-intensive Limited control over microstructure and active material loading High variability in performance consistency Requires separate equipment for each step Limited design flexibility for complex shapes
3D Printing (AM)	Simplifies fabrication with a single setup Reduces time and labor Precise control over microstructure and material loading Enhances performance consistency Minimal waste, eco-friendly Enables complex, customizable geometries

**Table 2:** Overview of MJ-printed structural components for LIBs.

Printed Structural Component		Ink Composition	The Electrochemical Properties	Printer	Refs Year
Cathode	LCO thin-film electrode	LCO (active cathode material) + AB (conducting agent) + CH10B/CH12B (polymeric hyper dispersants) + SCMC (binder). Mixed solvent was used.	Initial discharge capacity of 81 mAhg^−1^ at a discharge current of 20 A/cm^2^ between 3.0 and 4.2 V; 87% capacity retention after 50 charge–discharge cycles.	Canon BJC-1000sp (Canon Inc., Tokyo, Japan)	[114] 2007
	Round-shaped thin-film LCO electrode with a printed layer thickness of 1.2 μm (30 printing bands)	300 mg LCO (active cathode material) + 10 mL deionized water (solvent) + 1.5 mL of a 2 mg/mL commercial surfactant solution + 15 mg of conductive carbon black + 1 mL of monoethanolamine (PH adjustment) + 1.5 mg of SCMC (binder).	Initial discharge capacity of 120 mAhg^−1^ at a discharge current density of 180 μA/cm^2^; 95% capacity retention after 100 charge–discharge cycles.	Canon BJC-1000sp (Canon Inc., Tokyo, Japan)	[43] 2008
	LCO thin-film electrode	93 wt. % of active cathode material, 3 wt. % CB and 4 wt. % PVDF binder.	The discharge capacities for the electrodes using conventional, UV/ozone, and UV/ozone–TETA-treated CB were 121.6, 128.2, and 140.8 mAhg^−1^, respectively.	An ink-jet printer (Fujifilm Dimatix Inc., Santa Clara, CA, USA)	[42] 2011
	LCO electrode	LCO + Cured binder + Super P (80:10:10 wt. %). Novel acrylate-based curable inks was used.	Initial discharge capacity of 147.8 mAhg^−1^ at a discharge current of 0.1C.	−	[115] 2018
	Printed LFP electrodes with a thickness of 20 μm, including the current collector	LFP/C (active cathode material) + CB (conductive agent) + SCMC (binder) (80:10:10 wt. %) + buffer solution (HCl + NaOH) used as solvent + triton X-100 (surfactant) + glycerin (viscosity adjustment).	Initial discharge capacities of 129.9 mAhg^−1^ and 151.3 mAhg^−1^ at a discharge current rate of 0.1C (1C = 150 mAhg^−1^), using Al and CNT paper current collectors, respectively.	Dimatix-2800 (Fujifilm Dimatix, Inc., Santa Clara, CA, USA)	[40] 2015
	LFP-based composite porous electrodes with a thickness of 4 μm (40 printing bands)	LFP/C (active cathode material) + CB (conductive agent) + PAMA (binder) (85:10:5 wt. %). Deionized water was used as a solvent.	Discharge capacities of 80 mAhg^−1^ at a current rate of 9C and 70 mAhg^−1^ at 90C using organic electrolyte. Discharge capacity of 63 mAhg^−1^ at a current rate of 9C using the ionic liquid-based electrolyte.	Piezoelectric ink-jet printer (Fujifilm Dimatix, Inc., Santa Clara, CA, USA)	[41] 2015
	Printed 1.20 NCM and 1.25 NCM electodes	Li-rich cathode active material + CB (conductive agent) + PVDF (binder) (80:10:10 wt. %). NMP was used as a solvent. Different additives such as ethylene glycol, diethylene glycol, propylene glycol were studied.	Both electrodes have a discharge capacity of more than 250 mAhg^−1^ at a current rate of 0.1C between 2.5 and 4.8V.	Dimatix DMP-2831 inkjet printer (Fujifilm Dimatix, Inc., Santa Clara, CA, USA)	[106] 2019
	LMR-K with a thickness of 7 μm	Li-rich cathode active material + CB (conductive agent) + PVDF (binder) (80:10:10 wt. %). NMP was used as a solvent.	Initial discharge capacity of 240 mAhg^−1^ at a current rate of 0.01C.	Dimatix DMP-2831 inkjet printer (Fujifilm Di-matix, Inc., Santa Clara, CA, USA)	[103] 2020
	2D heterostructure electrode V_2_O_5_ /MXene	V_2_O_5_: conductive agent: binder = 70:20:10 wt. %. A mass ratio of 80:20 for V_2_O_5_/ Ti_3_C_2_T_x_ was used.	Initial discharge capacity of 321 mAhg^−1^ at a current rate of 1C; 91.8% capacity retention after 680 charge–discharge cycles.	Dimatix DMP-2800 inkjet printer (Fujifilm Dimatix, Inc., Santa Clara, CA, USA)	[108] 2021
Solid Electrolyte	Li_7_La_3_Zr_2_O_12_ solid electrolyte	LLZ active material + *n*-butanol + alpha-terpineol + PVB binder + BBP plasticizer (30% solid loading).	Average overpotential about 2.3 mV at a current density of 0.1 mAcm^−2^; area-specific resistance of 22 Ω cm^−2^.	nScrypt 3Dn-300 printer (Tabletop-3Dn, nScrypt Inc., Orlando, FL, USA)	[116] 2018
Full cell	3D interdigitated microbattery architectures (3D-IMA)	LTO/LFP + glycerol, ethylene glycol, hydroxypropyl cellulose, hydroxyethyl cellulose (solid loading: 55-65 wt. %).	The areal capacity of the packaged 3D-IMA was 1.2 mAhcm^−2^ at a current rate of 0.5C.	−	[44] 2013
	Fully 3D printed and packaged LIBs	Cathode: 30 vol. % LFP + 1.25 vol. % KB (conductive agent) + 1 wt. % PVP (nonionic dispersant) + LiTFSI/PC; Anode: 30 vol. % LTO + 1.35 vol. % KB (conductive agent) + 1 wt. % PVP (nonionic dispersant) + LiTFSI/PC; Separator: PC + triton TX-100 + Al_2_O_3_ + HMMP solution + LiTFSI/PC; Package: SiO_2_ + epoxy (4:96 vol. %).	The areal capacity of 4.45 mAhcm^−2^ at a current density of 0.14 mAcm^−2^.	Custom-made 3D printer	[117] 2018
		Cathode: LFP/rGO (70:30 wt. %); Anode: LTO/rGO (70:30 wt. %); Electrolyte: PVDF-co-HFP + Al_2_O_3_.	~100 mAhg^−1^ at 50 mAg^−1^.	-	[109] 2016

Abbreviations used in the table: AB—acetylene black; BBP—benzyl butyl phthalate; CB—carbon black; CMC—carboxymethyl cellulose; CNT—carbon nanotubes; HMMP—hexamethylenetetramine; KB—Ketjenblack (a very pure carbon black); LCO—LiCoO_2_; LFP—LiFePO_4_; LLZ—Li_7_La_3_Zr_2_O_12_; LMR-K—Li_1.15_K_0.05_Mn_0.54_Ni_0.13_Co_0.13_O_2_; 1.20 NCM—Li_1.2_Mn_0.54_Ni_0.13_Co_0.13_O_2_; 1.25 NCM—Li_1.25_Mn_0.54_Ni_0.13_Co_0.13_O_2_; LiTFSI/PC—lithium bis(trifluoromethanesulfonyl)imide (LiTFSI) dissolved in propylene carbonate (PC); NMP—N-methyl-2-pyrrolidone; PAMA—poly-acrylic-co-maleic acid; PEDOT:PSS—poly(3,4-ethylenedioxythiophene) polystyrene sulfonate; PVDF—polyvinylidene fluoride; PVDF-co-HFP—copolymer of PVDF and hexafluoropropylene (HFP); PVB—polyvinyl butyral; PVP—polyvinylpyrrolidone; rGO—reduced graphene oxide; SCMC—sodium carboxymethyl cellulose.

**Table 3:** Comparison of three representative printing techniques applied in the fabrication of LIBs.

Printing Technique	Resolution (µm)	Advantages	Limitations
Material Extrusion	50–200 (FDM) 1–250 (DIW)	Cost-effective: Suitable for lower-cost production. Versatility: Can handle a wide variety of materials, including thermoplastics and composites. Scalability: Effective for bulk deposition of simple structures, but scalability is hindered by speed.	Resolution: Struggles to achieve satisfactory resolution for complex microstructures. Speed: Slow process, which is a significant drawback for large-scale and commercial production. Material Constraints: Limited materials that meet battery electrode properties (conductivity).
Material Jetting	5–200 (IJP)	High Precision: Enables the creation of fine structures with minimal material wastage. Functional Materials: Can deposit conductive inks without binders, maintaining electrochemical performance. Customization: Ideal for highly customized electrode geometries with tailored material properties.	Material Compatibility: Not all materials are compatible, requiring adjustment of viscosity. Scalability: Limited when dealing with large electrode areas or batch production. Post-Processing: This may require additional curing or treatment steps to reach optimal performance.
Vat Photopolymerization	10–25 (SLA)	High Resolution: SLA offers the highest resolution among the methods for detailed structures. Complex Geometries: Capable of producing intricate electrode structures, enhancing electrochemical performance. Material Flexibility: Allows customization of material properties with a variety of photopolymerizable resins.	Material Limitations: Restricted range of materials that meet mechanical and conductive needs. Limited Build Volume: Smaller build volume compared to ME and MJ, limiting large-scale production. Curing and Post-Processing: Requires additional curing steps, increasing production complexity.

## Data Availability

No new data were created or analyzed in this study.
